# Comparison of Clinical Characteristics for Distinguishing COVID-19 From Influenza During the Early Stages in Guangdong, China

**DOI:** 10.3389/fmed.2021.733999

**Published:** 2021-11-11

**Authors:** Yongzhi Li, Huan He, Yuhan Gao, Zejin Ou, Wenqiao He, Caiyun Chen, Jiaqi Fu, Husheng Xiong, Qing Chen

**Affiliations:** Guangdong Provincial Key Laboratory of Tropical Disease Research, Department of Epidemiology, School of Public Health, Southern Medical University, Guangzhou, China

**Keywords:** COVID-19, influenza, retrospective cohort study, clinical characteristics, ROC curve

## Abstract

**Background:** To explore the differences in clinical manifestations and infection marker determination for early diagnosis of coronavirus disease-2019 (COVID-19) and influenza (A and B).

**Methods:** A hospital-based retrospective cohort study was designed. Patients with COVID-19 and inpatients with influenza at a sentinel surveillance hospital were recruited. Demographic data, medical history, laboratory findings, and radiographic characteristics were summarized and compared between the two groups. The chi-square test or Fisher's exact test was used for categorical variables, and Kruskal–Wallis *H*-test was used for continuous variables in each group. Receiver operating characteristic curve (ROC) was used to differentiate the intergroup characteristics. The Cox proportional hazards model was used to analyze the predisposing factors.

**Results:** About 23 patients with COVID-19 and 74 patients with influenza were included in this study. Patients with influenza exhibited more symptoms of cough and sputum production than COVID-19 (*p* < 0.05). CT showed that consolidation and pleural effusion were more common in influenza than COVID-19 (*p* < 0.05). Subgroup analysis showed that patients with influenza had high values of infection and coagulation function markers, but low values of blood routine and biochemical test markers than patients with COVID-19 (mild or moderate groups) (*p* < 0.05). In patients with COVID-19, the ROC analysis showed positive predictions of albumin and hematocrit, but negative predictions of C-reactive protein (CRP), procalcitonin (PCT), lactate dehydrogenase (LDH), hydroxybutyrate dehydrogenase (HBDH), and erythrocyte sedimentation rate. Multivariate analysis revealed that influenza might associate with risk of elevated CRP, PCT, and LDH, whereas COVID-19 might associated with high HBDH.

**Conclusion:** Patients with influenza had more obvious clinical symptoms but less common consolidation lesions and pleural effusion than those with COVID-19. These findings suggested that influenza likely presents with stronger inflammatory reactions than COVID-19, which provides some insights into the pathogenesis of these two contagious respiratory illnesses.

## Introduction

Influenza is one of the most common infectious respiratory diseases and has a high morbidity, resulting in 500,000 deaths annually worldwide (1, 2). Influenza is caused by virus types A, B, and C (3). Seasonal flu epidemics is attributed to the influenza A and B viruses spread routinely each year (4, 5). Influenza can cause mild-to-severe illness and even death. Symptoms range from fever, sore throat, runny nose, cough, headache, muscle pain, and fatigue to severe and in some cases lethal pneumonia (3, 6). Timely diagnosis and treatment of severe influenza can help reduce mortality (7, 8).

Coronavirus disease-2019 (COVID-19) is a novel respiratory infection caused by severe acute respiratory syndrome coronavirus 2 (SARS-CoV-2) (9). It was first reported in late December 2019, and until September, there were approximately 219 million confirmed cases and over 4.5 million deaths reported to the WHO (10–12). Serological studies showed that 263.5 million individuals at the time of this study had been exposed or infected by SARS-CoV-2, extrapolating to the 2020 world population (13). Influenza activity began to decline during the COVID-19 pandemic in both the Northern and Southern Hemispheres (14), suggesting that COVID-19 may also become a seasonal infectious disease in the future like influenza. COVID-19 can cause mild-to-severe illness, which is similar to influenza. Most patients with COVID-19 have clinical self-limited manifestations such as fever, cough, and myalgia or fatigue, which cannot be easily distinguished from those of influenza (15–17). Research on the clinical features and pathogenic mechanisms for COVID-19 is ongoing. We considered that it is important to understand clinical features of diseases. Not only the clinical diagnosis needs, the clinical symptoms also may reflect the pathogenic mechanism. Because the symptoms and signs associated with infectious disease are actually direct manifestations of the immune responses of the host in action (18).

So far, 12 original studies have been reported differences in the clinical characteristics between COVID-19 and influenza. Two studies reported that patients with COVID-19 had a higher mortality and worse clinical outcomes than patients with influenza, but these two reports lacked a comparison of clinical symptoms and radiography of the two conditions (19, 20). Two studies were conducted in pediatric population and also compared influenza A pneumonia with COVID-19 pneumonia (21, 22). Another two studies, in which the sample sources of the two diseases are not from the same areas (22, 23). Three comparative studies focused on acute respiratory distress syndrome (ARDS) patients with COVID-19 and influenza A (H1N1) (24–26). Only two publications compared influenza B with COVID-19 (27, 28). As far as we know, currently, there is a lack of comparative studies between influenza (A and B) and COVID-19 in adult patients.

Herein, we conducted a hospital-based retrospective cohort study to compare the clinical symptoms, signs, radiography, and peripheral blood indicators of influenza (A and B) and COVID-19. The study was implemented in a sentinel surveillance hospital for influenza and a designated hospital for COVID-19. We hope that the study results will provide a more comprehensive understanding for accurate distinction between the two diseases.

## Methods

### Study Design

This was a hospital-based retrospective cohort study conducted in April 2020. Two cohorts of hospitalized patients were employed for this study: COVID-19 cohorts and influenza cohorts. The patients hospitalized at a National Influenza Surveillance Hospital in the west of Guangdong province (China) between January 1, 2019 and April 10, 2020. Diagnoses were confirmed by laboratory tests.

### Data Collection

Demographic and clinical data of the patients were entered into an electronic case report form. The data included the following: demographic characteristics (age and sex), medical history (underlying diseases and epidemiological exposure), clinical symptoms (fever, cough, sputum production, weakness, shortness of breath, sore muscles, sore throat, headache, chest pain, chills, nasal obstruction, runny nose, nausea, vomiting and diarrhea), body signs (body temperature, pulse rate, respiratory rate, and blood pressure), laboratory tests (routine blood tests, coagulation function, biochemical tests, and inflammatory index), and pulmonary images (chest CT scan). Complications and outcomes were also recorded. The specific definition and reference range of variables were given in Supplementary Tables 1, 2.

Diagnoses of COVID-19 or influenza were based on clinical presentations, imaging characteristics, and the presence of either SARS-CoV-2 or influenza viruses detected in samples from either the respiratory tract or blood. Three types of COVID-19 infection were classified in our study: mild, moderate, and severe based on the Guidelines for the Diagnosis and Treatment of COVID-19 Infection by the National Health Commission (Trial Version 5) of China (29).

### Statistical Analysis

Data analysis was performed using the SPSS software (version 26.0; IBM Corporation, Armonk, NY, USA). Propensity score matching based on the nearest neighbor matching method was used in two cohorts. Predictors were age and sex with match tolerance 0.02. Categorical variables were summarized using frequencies and percentages, and continuous data were presented as the medians [interquartile ranges (IQRs)]. The chi-square test or Fisher's exact test was used for categorical variables, and Kruskal–Wallis *H*-test was used for continuous variables for each group. Receiver operating characteristic (ROC) curve was used to analyze the diagnostic efficacy of peripheral biochemical parameters. We performed the Cox proportional hazards model for analyzing predisposing factors. The time interval between first visit and definite diagnosis was included as a time variable. All *p-*values <0.05 were considered to indicate statistical significance.

## Results

About 23 cases with COVID-19 were admitted to a sentinel surveillance hospital in the west of Guangdong province (Supplementary Table 1). There were 591 patients with influenza virus A (H1N1) or influenza virus B admitted to the same hospital from January 1, 2019 to April 10, 2020. Among them, 74 patients (59 patients with influenza-A and 15 patients with influenza-B) were matched with the patients COVID-19 for this study.

### Demographic Characteristics Between COVID-19 and Influenza Groups

**Figure 1:**
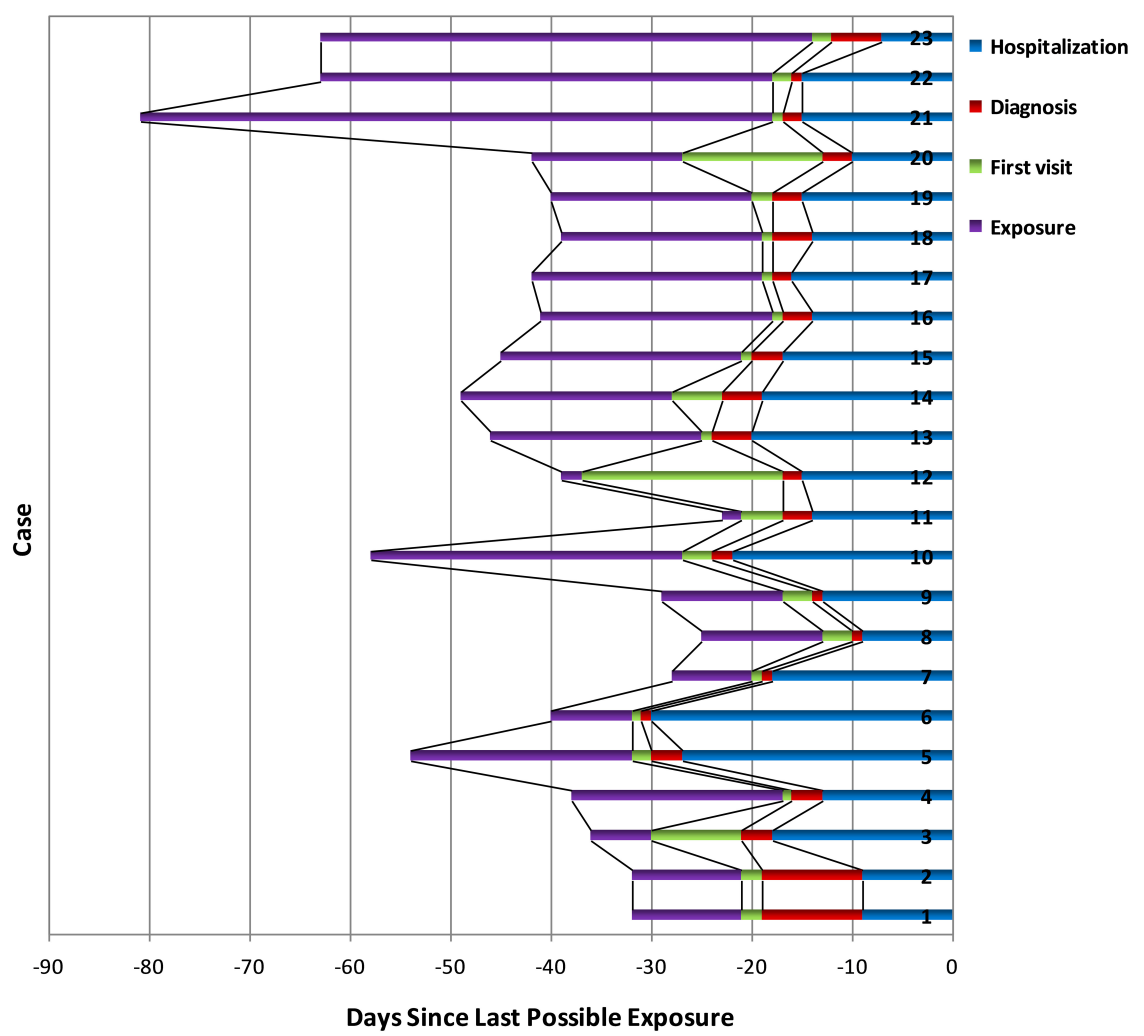
SARS-CoV-2 exposure (*purple*), first visit (*green*), definite diagnosis (*red*), and hospitalization time (*blue*) for 23 confirmed cases. Bar regions represented the full possible time intervals for exposure, first visit, definite diagnosis, and hospitalization; serial number of cases was arranged according to the order of incidence.

The median age of patients with influenza-A was 46.0 (29.0–58.0) years, whereas the median age of patients with influenza-B was 34.0 (21.0–57.0) years (Tables 1, 2). Among the patients with COVID-19, 87.0% were aged between 15 and 64 years, with a median age of 33.0 (25.0–51.0) years (Supplementary Table 1). The proportion of male-to-female subjects was comparable between the COVID-19 and influenza groups (*p* = 0.634 and *p* = 0.564, respectively) (Supplementary Tables 1, 2). Epidemiological exposure history showed that 39.1% patients were indigenous cases, five coming from Wuhan, and three from abroad in 23 COVID-19 cases (Supplementary Table 1). The incubation period for those patients with COVID-19 who came back from overseas was prolonged (Figure 1). Half the patients with influenza-A had at least one underlying illness, but 82.6% of the patients with COVID-19 had no underlying disease (*p* = 0.012).

**Table 1:** Characteristics of patients with COVID-19 or influenza A.

**Characteristics**	**Total (*n* = 82)**	**COVID−19 (*n* = 23)**	**Influenza A (*n* = 59)**	***p-*value**
Age (years, IQR)	42.0 (27.0–58.0)	33.0 (25.0–51.0)	46.0 (29.0–58.0)	0.086
Male (%)	41 (50.0)	13 (56.5)	28 (47.5)	0.624
Median time interval between first visit, days (IQR)	1.0 (0–3.0)	1.0 (0–3.0)	1.0 (0–3.0)	0.625
Duration of fever after admission, days (IQR)	2.0 (0.8–2.0)	1.0 (0–2.0)	1.0 (0–2.0)	0.578
Body temperature (>37°C, %)	30 (36.6)	10 (43.5)	20 (33.9)	0.452
Abnormal blood pressure (mmHg, %)	19 (23.2)	3 (13.0)	16 (27.1)	0.247
Respiratory rate (>20/min, %)	20 (24.4)	3 (13.0)	17 (28.8)	0.163
Pulse rate (>100/min, %)	19 (23.2)	0	19 (32.2)	**0.001**
Underlying disease (%)				**0.012**
None	46 (56.1)	19 (82.6)	27 (45.8)	
1 kind	16 (19.5)	1 (4.3)	15 (25.4)	
2 kinds	14 (17.1)	1 (4.3)	13 (22.0)	
3 kinds	6 (7.3)	2 (8.7)	4 (6.8)	
**Infection with other respiratory pathogens (%)**				**0.000**
*Chlamydia*/*Mycoplasma pneumoniae*	3 (3.7)	2 (8.7)	1 (1.7)	0.389
*Legionella pneumophila*	4 (4.9)	3 (13.0)	1 (1.7)	0.116
*Rickettsia*	1 (1.2)	0	1 (1.7)	1.000
**Hepatic or renal insufficiency (%)**	9 (15.3)	0	9 (11.0)	0.056

*IQR, interquartile range. Bold values indicate statistically significant*.

**Table 2:** Characteristics of patients with COVID-19 or influenza B.

**Characteristics**	**Total (*n* = 38)**	**COVID−19 (*n* = 23)**	**Influenza B (*n* = 15)**	***p-*value**
Age (years, IQR)	33.5 (24.0–53.8)	33.0 (25.0–51.0)	34.0 (21.0–57.0)	0.858
Male (%)	22 (57.9)	13 (56.5)	9 (60.0)	0.832
Median time interval between first visit, days (IQR)	1.0 (0–4.0)	1.0 (0–3.0)	1.0 (0–3.8)	0.096
Duration of fever after admission, days (IQR)	1.0 (0–2.0)	1.0 (0–2.0)	1.0 (0–2.0)	0.723
Body temperature (>37°C, %)	14 (36.8)	10 (43.5)	4 (26.7)	0.329
Abnormal blood pressure (mmHg, %)	5 (13.2)	3 (13.0)	2 (13.3)	0.979
Respiratory rate (>20/min, %)	6 (15.8)	3 (13.0)	3 (20.0)	0.663
Pulse rate (>100/min, %)	2 (5.3)	0	2 (13.3)	0.149
Underlying disease (%)				0.138
None	27 (71.1)	19 (82.6)	8 (53.3)	
1 kind	5 (13.2)	1 (4.3)	4 (26.7)	
2 kinds	3 (7.9)	1 (4.3)	2 (13.3)	
3 kinds	3 (7.9)	2 (8.7)	1 (6.7)	
**Infection with other respiratory pathogens (%)**				**0.000**
*Chlamydia*/*Mycoplasma pneumoniae*	4 (10.5)	2 (8.7)	2 (13.3)	1.000
*Legionella pneumophila*	3 (7.9)	3 (13.0)	0	0.400
*Rickettsia*	1 (2.6)	0	1 (6.7)	0.827
**Hepatic or renal insufficiency (%)**	4 (10.5)	0	4 (26.7)	**0.018**

*IQR, interquartile range. Bold values indicate statistically significant*.

For patients with COVID-19, infection rates with *Legionella pneumophila* and *Chlamydia*/*Mycoplasma pneumoniae* were 13.0 and 8.7%, respectively, which were higher than that for patients with influenza-A (*p* < 0.001, Table 1). However, *Chlamydia* or *Mycoplasma pneumoniae* co-infection in patients with influenza-B was more frequent than in patients with COVID-19 (13.3 vs. 8.7%, *p* < 0.001, Table 2). In addition, 11% patients with influenza-A and 26.7% patients with influenza-B presented hepatic or renal insufficiency compared to COVID-19 (0%, Table 2).

### Clinical Symptoms of Subjects

#### Clinical Signs

Both influenza and patients with COVID-19 presented fever, cough, sputum production, shortness of breath, chills, sore throat, and weakness (Tables 3, 4). For patients with influenza-A or influenza B, proportions of cough and spontaneous sputum production were both significantly higher than for patients with COVID-19 (*p* < 0.05, Tables 3, 4). Similarly, 33.3% patients with influenza-B presented symptoms of headache, which was significantly higher than in patients with COVID-19 (*p* = 0.006, Table 4). However, patients with COVID-19 had obvious symptoms of weakness than those with influenza (both A and B).

**Table 3:** Clinical symptoms of patients with COVID-19 and influenza-A.

**Characteristics**	**Total (*n* = 82)**	**COVID-19 (*n* = 23)**	**Influenza A (*n* = 59)**	***p-*value**
**Clinical signs (%)**				
Fever	71 (86.6)	18 (78.3)	53 (89.8)	0.277
Cough	70 (85.4)	11 (47.8)	59 (100.0)	**0.000**
Sputum production	60 (73.2)	9 (39.1)	51 (86.4)	**0.000**
Weakness	13 (15.9)	8 (34.8)	5 (8.5)	**0.006**
Shortness of breath	30 (36.6)	8 (34.8)	22 (37.3)	0.832
Sore muscles	2 (2.4)	1 (4.3)	1 (1.7)	0.485
Sore throat	14 (17.1)	4 (17.4)	10 (16.9)	0.962
Headache	10 (12.2)	0	10 (16.9)	0.055
Chest pain	5 (6.1)	0	5 (8.5)	0.315
Chills	23 (28.0)	4 (17.4)	19 (32.2)	0.274
Nasal obstruction	8 (9.8)	2 (8.7)	6 (10.2)	0.840
Runny nose	9 (11.0)	3 (13.0)	6 (10.2)	0.705
Nausea	6 (7.3)	2 (8.7)	4 (6.8)	0.765
Vomiting	6 (7.3)	1 (4.3)	5 (8.5)	0.519
Diarrhea	8 (9.8)	4 (17.4)	4 (6.8)	0.146
**Radiographic characteristics (%)**				
Ground-glass opacities	7 (8.5)	7 (30.4)	0	**0.000**
Consolidation and pleural effusion	18 (22.0)	0	18 (30.5)	**0.002**

*Bold values indicate statistically significant*.

**Table 4:** Clinical symptoms of patients with COVID-19 and influenza-B.

**Characteristics**	**Total (*n* = 38)**	**COVID-19 (*n* = 23)**	**influenza B (*n* = 15)**	***p-*value**
**Clinical signs (%)**				
Fever	30 (78.9)	18 (78.3)	12 (80.0)	0.898
Cough	25 (65.8)	11 (47.8)	14 (93.3)	**0.005**
Sputum production	22 (57.9)	9 (39.1)	13 (86.7)	**0.006**
Weakness	11 (28.9)	8 (34.8)	3 (20.0)	0.470
Shortness of breath	14 (36.8)	8 (34.8)	6 (40.0)	0.744
Sore muscles	4 (10.5)	1 (4.3)	3 (20.0)	0.280
Sore throat	6 (15.8)	4 (17.4)	2 (13.3)	0.737
Headache	5 (13.2)	0	5 (33.3)	**0.006**
Chest pain	0	0	0	/
Chills	10 (26.3)	4 (17.4)	6 (40.0)	0.122
Nasal obstruction	4 (10.5)	2 (8.7)	2 (13.3)	0.649
Runny nose	3 (7.9)	3 (13.0)	0	0.264
Nausea	5 (13.2)	2 (8.7)	3 (20.0)	0.365
Vomiting	3 (7.9)	1 (4.3)	2 (13.3)	0.550
Diarrhea	5 (13.2)	4 (17.4)	1 (6.7)	0.630
**Radiographic characteristics (%)**				
Ground-glass opacities	7 (18.4)	7 (30.4)	0	**0.029**
Consolidation and pleural effusion	4 (10.5)	0	4 (26.7)	**0.018**

*Bold values indicate statistically significant*.

#### Radiographic Characteristics

**Figure 2:**
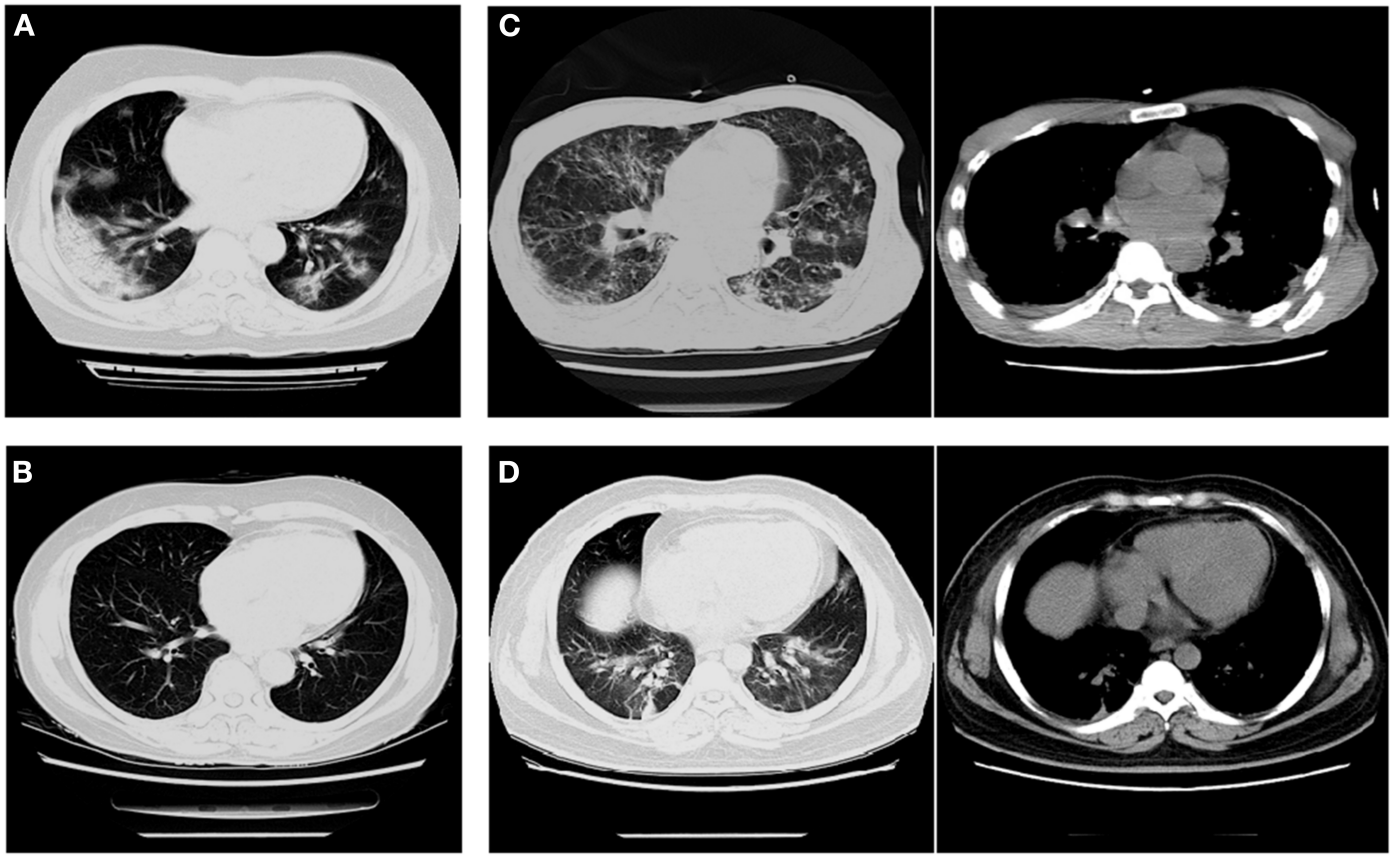
Imaging characteristics of chest CT scans from patients with COVID-19 and influenza pneumonia. **(A)** A 62-year-old woman with COVID-19 (critical illness) exhibited multiple ground-glass opacities in both lungs. **(B)** A 63-year-old woman with influenza-B exhibited fibrous indurations in the upper left and lower right lungs. **(C)** A 47-year-old man with H1N1 exhibited pleural effusion in the medial posterior chest wall and nodules distributed within the lobar bronchus. **(D)** A 45-year-old man with influenza-A exhibited a small amount of pleural effusion and the texture of both lungs was increased and thickened.

Among patients with COVID-19, 30.4% showed ground-glass opacity (GGO) on the chest CT compared with patients with influenza (A and B) (Tables 3, 4, Figure 2A). However, consolidation and pleural effusion on chest CTs were more common in patients with influenza (A and B) (30.5% and 26.7%, respectively; *p* < 0.05; Tables 3, 4, Figures 2B–D).

### Laboratory Biochemical Examinations

#### Routine Blood Tests

The percentage of neutrophilic granulocytes was 72.0 (59.2–81.4) in patients with influenza-A, which was dramatically higher than that in patients with COVID-19 (*p* < 0.001, Supplementary Table 3). Although the lymphocyte count and lymphocyte percentage decreased in both patients with influenza-A and COVID-19, patients with influenza-A had a drastically larger decline than patients with COVID-19 (*p* < 0.001). For patients with influenza-A, the red blood cell (RBC) count was 3.8 (3.4–4.7) × 10^12^/L, which was significantly lower than the RBC count of patients with COVID-19 [4.7 (4.4–5.1) × 10^12^/L, *p* = 0.001, Supplementary Table 3]. However, there was an obvious difference in the white blood cell (WBC) count between influenza-B and patients with COVID-19 (8.8 × 10^9^/L vs. 6.6 × 10^9^/L, *p* = 0.010, Supplementary Table 4). The values of hemoglobin (Hgb) and hematocrit (Hct) declined sharply in both patients with influenza (A and B) and COVID-19 (*p* < 0.05, Supplementary Table 3). Nevertheless, the Hct of patients with influenza-B was 34.1 (31.1–39.9) and lower than that of patients with COVID-19 (*p* = 0.002, Supplementary Table 4).

#### Coagulation Function

Both patients with influenza and COVID-19 had impaired coagulation function. The values of D-dimer in patients with influenza-A (0.61 mg/L) and influenza-B (0.74 mg/L) were both significantly higher than those in patients with COVID-19 (0.22 mg/L, *p* < 0.05, Supplementary Tables 3, 4). The erythrocyte sedimentation rate (ESR) in both patients with influenza and COVID-19 climbed up drastically, while the increases in patients with influenza (influenza-A: 36.5 mm/h and influenza-B: 42.0 mm/h) were higher than those in patients with COVID-19 (22.0 mm/h, *p* < 0.001, Supplementary Tables 3, 4).

#### Biochemical Tests

The levels of total protein (TP) and albumin (Alb) in patients with influenza (A and B) were significantly lower than those in patients with COVID-19 (*p* < 0.05, Supplementary Tables 3, 4). However, the levels of aspartate transaminase (AST), hydroxybutyrate dehydrogenase (HBDH), and lactate dehydrogenase (LDH) in patients with influenza (A and B) were all obviously higher than those in patients with COVID-19 (*p* < 0.05, Supplementary Tables 3, 4). In addition, the level of gamma-glutamyl transferase (GGT) in patients with influenza-B was significantly higher than in patients with COVID-19, but there was no big difference between patients with influenza-A and COVID-19 (Supplementary Tables 3, 4).

#### Inflammatory Index

The level of C-reactive protein (CRP) was 25.0 mg/L in patients with influenza-A, which was five times higher than that in patients with COVID-19 (*p* < 0.001, Supplementary Table 3). The comparison of CRP between patients with influenza-B and COVID-19 showed similar results (26.1 mg/L vs. 4.8 mg/L, *p* < 0.05, Supplementary Table 4). Furthermore, patients with both influenza-A (0.23 ng/mL) and influenza-B (0.11 ng/mL) had higher levels of procalcitonin (PCT) than patients with COVID-19 (0.03 ng/mL, *p* < 0.05, Supplementary Tables 3, 4).

### Subgroup Analysis Between COVID-19 and Influenza Groups

We performed subgroup analysis according to the classification of three types of patients with COVID-19 in our study: mild, moderate, and severe. The results showed that there was no great difference in age, sex, and underlying disease between the influenza and COVID-19 subgroups (*p* > 0.05, Supplementary Table 5). However, for patients with influenza, symptoms of fever, cough, sputum production, and inflammatory indices of CRP, PCT, D-dimer, ESR, and fibrinogen content were higher, to a great extent, than the mild and moderate groups of COVID-19 (*p* < 0.05, Supplementary Table 5).

### Diagnostic Efficacy of Peripheral Biochemical Parameters Between the COVID-19 and Influenza Groups

**Figure 3:**
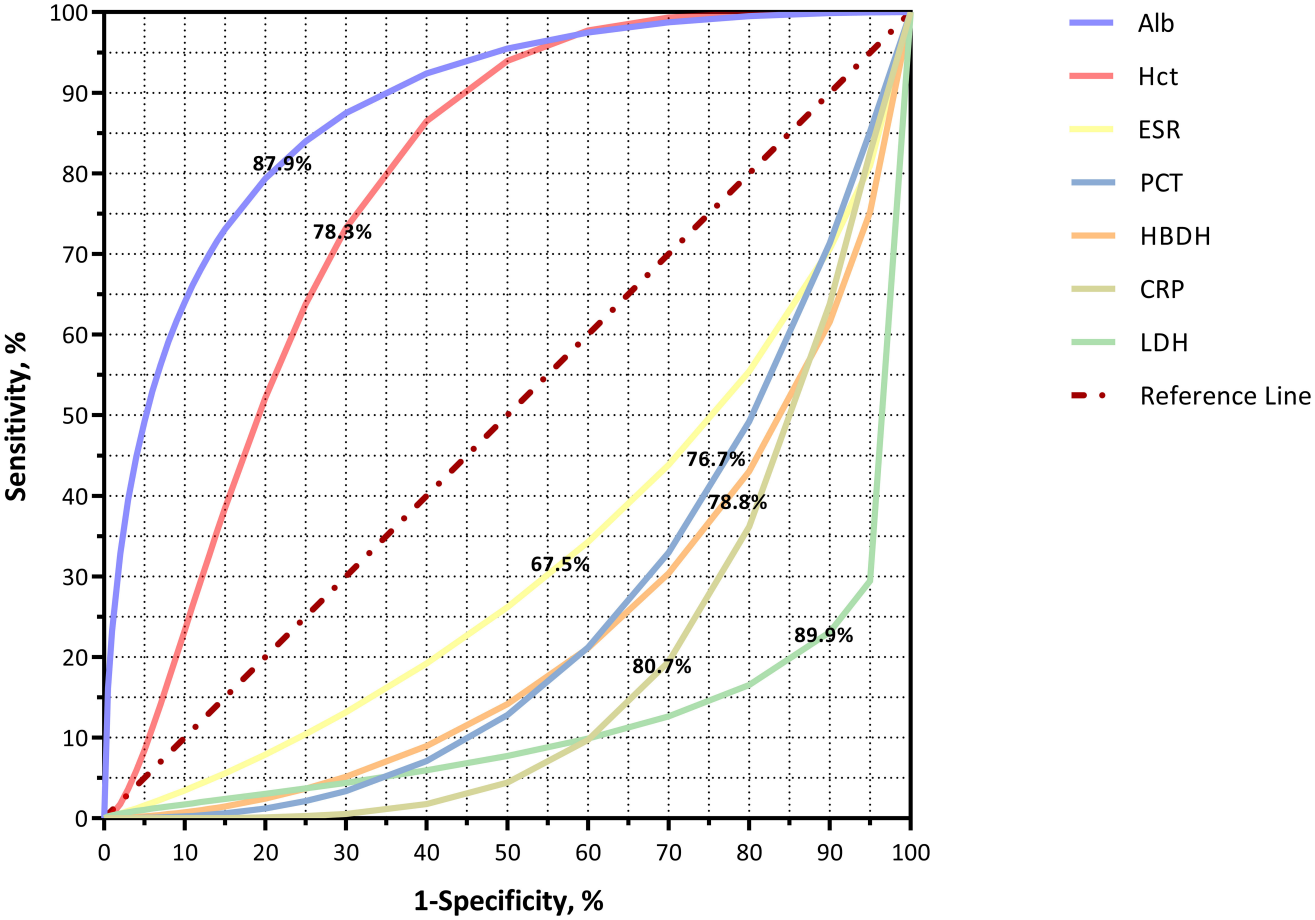
ROC curves for differentiating COVID-19 from influenza patients.

COVID-19 was set as the positive group, and influenza (A and B) was set as the negative group. The ROC curve was used to make positive and negative predictions, suggesting that increases in Alb and Hct but decreases in CRP, PCT, LDH, HBDH, and ESR were more susceptible to COVID-19 (*p* < 0.001, Figure 3).

### Multivariate Analysis Between COVID-19 and Influenza Groups

**Figure 4:**
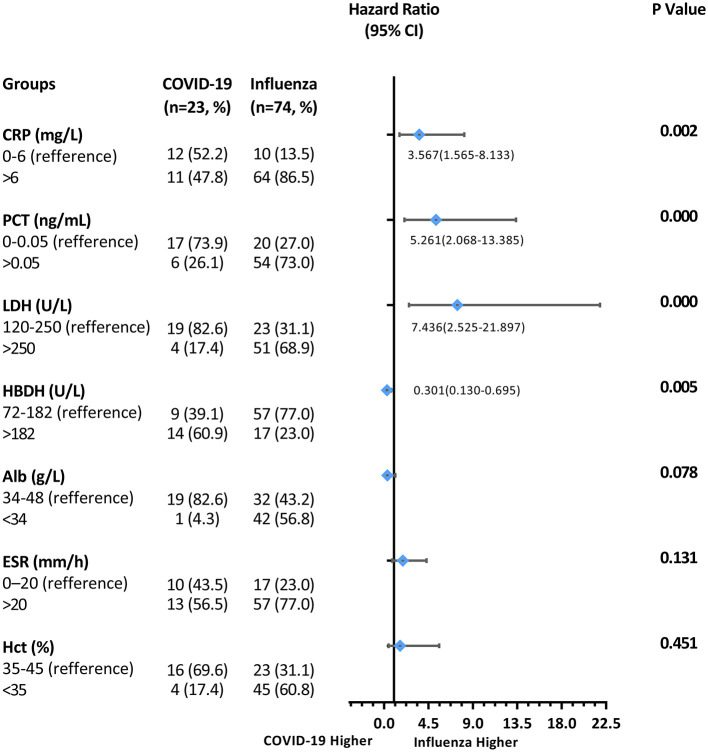
Multivariate analysis for differentiating COVID-19 from influenza patients.

Variables with a *p*-value <0.05 in the univariate analysis were included in the Cox proportional hazards model. Covariates of “cough,” “sputum production,” “Hgb,” “D-dimer,” and “Alb” were adjusted. Compared to patients with COVID-19, patients with influenza (A and B) showed a higher frequency of elevated CRP (hazard ratio, HR = 3.567, 95%CI: 1.565–8.133, *p* < 0.05, Figure 4); PCT (HR = 5.261, 95%CI: 2.068–13.385, *p* < 0.001); and LDH (HR = 7.436, 95%CI: 2.525–21.897, *p* < 0.001), but a lower frequency of elevated HBDH (HR = 0.301, 95%CI: 0.130–0.695, *p* = 0.005).

## Discussion

To reduce information bias, we selected patients with COVID-19 and influenza admitted to the same surveillance hospital. However, the number of COVID-19 cases of this administrative region, which were all included in our study, was only 23. Considering this limitation, we carried out propensity score matching and increased the matched cases of each influenza group to improve the statistical power and reliability of the study.

Our research revealed that the onset of both influenza and COVID-19 is characterized by fever, cough, sputum production, weakness, and systemic malaise. Patients with influenza tended to have respiratory symptoms, such as cough, sputum production, headache, chills, and high fever, which was similar to previous studies (30, 31). In the present study, local and systemic symptoms in patients with COVID-19 were milder than in those with influenza. The proportion of hepatic or renal insufficiency in patients with influenza was bigger than that of the patients with COVID-19 (*p* < 0.05). Nevertheless, two retrospective studies in France and the United States, respectively, both showed that patients with influenza presented more frequently with liver impairment than patients with COVID-19 (20, 25), but patients with COVID-19 with severe pneumonia had a higher frequency of kidney disease than influenza pneumonia (23).

We found that patients with influenza B showed significantly higher WBC counts than patients with COVID-19 (*p* < 0.05), but there was no statistical difference between patients with influenza A and COVID-19. The results were similar to previous studies that had more frequency in increased WBC levels (23, 31). The Alb of patients with influenza (A and B) was apparently lower than in patients with COVID-19 in our research, which was consistent with other studies in China (24, 31). Subgroup analysis showed that patients with influenza presented high CRP, PCT, D-dimer, ESR, and fibrinogen content, but low lymphocyte count, lymphocyte percentage, RBC, Hgb, Hct, TP, and Alb than patients with COVID-19 (mild or moderate groups) (*p* < 0.05), which was consistent with a previous study (31). So far, among published studies, there is few article displaying subgroup analysis of the two diseases according to the illness severity (32). Similarly, the above study (32) showed that the influenza group presented lower lymphocytes count, lymphocyte percentage, and RBC when compared to the common COVID-19 group, but no significant difference in Hgb.

The ROC curve was used to make positive and negative predictions, suggesting that increases in Alb and Hct but decreases in CRP, PCT, LDH, HBDH, and ESR were more susceptible to COVID-19 (Figure 3). However, the study in Hubei (27) showed a controversial result; in that, the proportion of increased CRP in patients with COVID-19 was significantly higher than in patients with influenza (*p* < 0.05), but there was no difference in the level of ESR. Similarly, the highest CRP was more frequently seen in patients with COVID-19 rather than in those with influenza in a Finnish study (33).

Multivariate analysis was performed using the Cox proportional hazards model. We found that patients with influenza had statistically higher levels of CRP, PCT, LDH, HBDH, and ESR, but lower levels of Alb and Hct on univariate analysis. However, only CRP, LDH, PCT, and HBDH were found to be statistically significant after multivariate analysis. The results showed that increased CRP, PCT, and LDH may associate with influenza (A and B) and increased HBDH with COVID-19.

Procalcitonin is a precursor of calcitonin that is constitutively secreted by the thyroid gland and lungs. PCT can serve as a severity index of pneumonia; lower PCT in COVID-19 may indicate less lung involvement (34). In previous studies, elevated LDH has a causal relationship with influenza (35, 36), similar to our results. The decreases of serum LDH are related to the elimination of viral messenger RNA (mRNA), with the COVID-19 viral mRNA clearance time positively correlated with the duration of hospital stay (37). Decreases in LDH may indicate a good prognosis for COVID-19. HBDH is a marker of cell death and an auxiliary marker of myocardial injury, reflecting renal, RBC, and myocardial damage (38). This was the first study to suggest that increased HBDH could be used to differentiate between COVID-19 and influenza. Existing literature has pointed out that elevated α- HBDH is an independent prognostic factor for mortality in hospitalized patients with COVID-19 (39).

Patients with COVID-19 showed plaque GGOs combined with turbidity, whereas the manifestations of influenza were thickening of the lung texture and symptoms of pleural effusion (40). A study based on the imaging findings of 919 patients with COVID-19 also reported that bilateral multi-lobe GGOs more often appeared in the early stage of the disease (41). However, a multicenter comparative study on differentiating COVID-19 from influenza in Wuhan and Zhenjiang found that there was no difference in location or distribution of pulmonary lesions for both viral infections, but patients with COVID-19 showed more diversity in CT patterns (22). In our study, the GGO in CT images was more common in patients with COVID-19 than patients with influenza. Patients with influenza presented pulmonary exudative lesions, which was consistent with a previous study and could cause pleural effusion in some cases (42). The clinical manifestations of COVID-19 were more concealed with fewer underlying diseases and slighter respiratory symptoms than influenza. The CT manifestations of COVID-19 pneumonia showed more GGO with a relatively clear margin, crazy-paving pattern, and early fibrotic lesions, but less common consolidation lesions and pleural effusion. Combining the different clinical and CT features is helpful to distinguish COVID-19 from influenza and gain a better understanding of both contagious respiratory illnesses.

The sample size was a limitation in our study. Future research in distinguishing COVID-19 from influenza should focus on well-designed, prospective studies with larger sample sizes to enable stronger evidence.

## Conclusions

Patients with influenza had more obvious clinical manifestations and common consolidation and pleural effusion than patients with COVID-19 in the early stages of the disease. These findings suggested that influenza (A and B) might present stronger inflammatory reactions than COVID-19, which likely provides some insights into the pathogenesis of the two contagious respiratory illnesses.

## Data Availability Statement

The original contributions presented in the study are included in the article/Supplementary Material, further inquiries can be directed to the corresponding author.

## Ethics Statement

The studies involving human participants were reviewed and approved by Human Ethics and Welfare Committee School of Public Health, Southern Medical University. Oral informed consent to participate in this study was provided by the participants' legal guardian/next of kin. Oral informed consent was obtained from the individual(s) for the publication of any potentially identifiable images or data included in this article.

## Author Contributions

YL and HH contributed to the project administration and drafting. YG and ZO performed data analysis and validation. WH and CC involved in data analysis and visualization. JF and HX helped in data collection and collation. QC supported in supervision, drafting, and editing. All authors contributed to the article and approved the submitted version.

## Conflict of Interest

The authors declare that the research was conducted in the absence of any commercial or financial relationships that could be construed as a potential conflict of interest.

## Publisher's Note

All claims expressed in this article are solely those of the authors and do not necessarily represent those of their affiliated organizations, or those of the publisher, the editors and the reviewers. Any product that may be evaluated in this article, or claim that may be made by its manufacturer, is not guaranteed or endorsed by the publisher.
