# Supplementary material for: Comparison of Clinical Characteristics for Distinguishing COVID-19 From Influenza During the Early Stages in Guangdong, China
Source: Front Med (Lausanne). 2021 Nov 11;8:733999. doi: 10.3389/fmed.2021.733999 (PMC8631935; doi:10.3389/fmed.2021.733999)
Supplement: Supplementary file 1 [file Data_Sheet_1.doc]

**Table S1. Demographic and clinical features of inpatients with COVID-19 stratified by three** clinical types

| **Characteristics** | **Total (n=23)** | **Mild Cases (n=7)** | **Moderate Cases (n=12)** | **Severe Cases (n=4)** | ***P* Value** | |
| --- | --- | --- | --- | --- | --- | --- |
| **Sex (%)**  Female  Male | 10(43.5)  13(56.5) | 2(28.6)  5(71.4) | 6(50.0)  6(50.0) | 2(50.0)  2(50.0) | 0.634 | |
| **Age, years (IQR)**  **Age, groups (%)**  0-14 years  15-49 years  50-64 years  ≥65 years | 33.0(25.0-51.0)  2(8.7)  14(60.9)  6(26.1)  1(4.3) | 24.0(10.0-37.0)  2(28.6)  4(57.1)  1(14.3)  0 | 36.0(26.0-51.0)  0  8(66.7)  3(25.0)  1(8.3) | 44.0(25.3-63.5)  0  2(50.0)  2(50.0)  0 | 0.191  0.330 | |
| **Underlying disease (%)**  None  1 kind  2 kinds  3 kinds | 19(82.6)  1(4.3)  1(4.3)  2(8.7) | 7(100)  0  0  0 | 9(75.0)  1(8.3)  1(8.3)  1(8.3) | 3(75.0)  0  0  1(25.0) | 0.672 | |
| **Epidemiological exposure history (%)**  Epidemic area (Wuhan)  Overseas tourism  Foreign students input  Indigenous cases | 6(26.1)  5(21.7)  3(13.0)  9(39.1) | 2(28.6)  2(28.6)  2(28.6)  1(14.3) | 2(16.7)  3(25.0)  1(8.3)  6(50.0) | 2(50.0)  0  0  2(50.0) | 0.425 | |
| **Family members with COVID-19 (%)** | 12(52.2) | 3(42.9) | 7(58.3) | 2(50.0) | 0.805 | |
| ***Symptoms (%)*** |  |  |  |  |  | |
| Fever | 18(78.3) | 3(42.9) | 11(91.7) | 4(100.0) | **0.023** | |
| Cough | 11(47.8) | 1(14.3) | 6(50.0) | 4(100.0) | **0.023** | |
| Sputum production | 9(39.1) | 1(14.3) | 4(33.3) | 4(100.0) | **0.017** | |
| Weakness | 8(34.8) | 0 | 6(50.0) | 2(50.0) | 0.068 | |
| Shortness of breath | 8(34.8) | 1(14.3) | 3(25.0) | 4(100.0) | **0.010** | |
| Sore muscles | 1(4.3) | 0 | 1(8.3) | 0 | 0.619 | |
| Sore throat | 4(17.4) | 1(14.3) | 3(25.0) | 0 | 0.503 | |
| Headache | 0 | 0 | 0 | 0 | / | |
| Chest pain | 0 | 0 | 0 | 0 | / | |
| Chills | 4(17.4) | 0 | 3(25.0) | 1(25.0) | 0.347 | |
| Nasal obstruction | 2(8.7) | 0 | 2(16.7) | 0 | 0.366 | |
| Runny nose | 3(13.0) | 0 | 2(16.7) | 1(25.0) | 0.429 | |
| Nausea | 2(8.7) | 2(28.6) | 0 | 0 | 0.082 | |
| Vomiting | 1(4.3) | 1(14.3) | 0 | 0 | 0.303 | |
| Diarrhea | 4(17.4) | 1(14.3) | 2(16.7) | 1(25.0) | 0.899 | |
| ***Body signs (at admission, %)*** |  |  |  |  |  | |
| Body temperature (>37℃) | 10(43.5) | 4(57.1) | 5(41.7) | 1(25.0) | 0.576 | |
| Pulse rate (>100 per minute) | 0 | 0 | 0 | 0 | / | |
| Respiratory rate (>20 per minute) | 3(13.0) | 1(14.3) | 0 | 2(50.0) | **0.036** | |
| Blood pressure (SBP>140mmHg) | 3(13.0) | 0 | 2(16.7) | 1(25.0) | 0.429 | |
| Duration of fever after admission, days (IQR) | 1.0(0-2.0) | 0 (0-2.0) | 1.0(0-2.0) | 3.5(0-9.3) | 0.892 | |
| ***Complications (%)*** |  |  |  |  |  | |
| **Infection with other respiratory pathogens**  *Chlamydia/Mycoplasma pneumoniae*  *Legionella pneumophila* | 2(8.7)  3(13.0) | 2(28.6)  0 | 0  3(25.0) | 0  0 | 0.106 | |
| ***Laboratory tests (reference values, IQR)*** |  |  |  |  |  | |
| White blood cells (4-10 × 10⁹ cells per L) | 6.6(5.6-7.9) | 5.9(5.6-8.3) | 6.1(5.5-6.8) | 8.2(7.1-8.8) | 0.058 | |
| Neutrophile granulocyte (1.8-6.3 × 10⁹ cells per L)  Increased (n) | 3.7(3.0-5.0)  2 | 3.3(1.8-5.0)  0 | 3.2(2.9-4.0)  0 | 5.8(5.0-6.7)  2 | **0.028** | |
| Neutrophilic granulocyte percentage (40-75%)  Decreased (n) | 56.8(49.2-64.5)  2 | 54.7(31.9-56.3)  2 | 56.8(50.0-62.0)  0 | 71.1(60.7-72.7)  0 | **0.020** | |
| Lymphocytes (1.1-3.2 × 10⁹ cells per L)  Decreased (n) | 1.9(1.6-2.6)  2 | 2.3(1.9-3.4)  0 | 2.0(1.5-2.5)  0 | 1.4(1.0-2.2)  2 | 0.085 | |
| Lymphocyte percentage (20-50%)  Decreased (n) | 33.1(25.9-40.7)  2 | 36.8(32.6-53.5)  0 | 33.8(27.1-39.5)  0 | 19.2(17.8-30.3)  2 | **0.043** | |
| Red blood cells (3.5-5 × 10^12^ cells per L)  Increased (n) | 4.7(4.4-5.1)  6 | 4.8(4.3-5.2)  2 | 4.6(4.4-5.0)  3 | 4.4(3.8-5.2)  1 | 0.765 | |
| Hemoglobin (110-150g/L)  Decreased (n) | 129.3(115.8-139.1)  5 | 123.7(115.8-141.9)  1 | 137.8(122.2-139.2)  2 | 115.9(102.0-126.0)  2 | 0.163 | |
| Hematocrit (Hct, 35-45%)  Increased (n)  Decreased (n) | 41.2(37.2-44.6)  3  4 | 39.8(37.2-44.6)  1  0 | 43.6(39.6-44.6)  2  2 | 36.2(32.9-40.1)  0  2 | 0.104 | |
| Platelet count (PLT, 100-300 × 10⁹ cells per L)  Increased (n) | 245.2(176.6-280.0)  4 | 273.5(188.0-302.8)  2 | 241.6(159.4-253.8)  1 | 245.4(164.9-366.4)  1 | 0.433 | |
| C-reactive protein (0-6 mg/L)  Increased (n) | 4.8(2.2-14.4)  11 | 2.2(1.7-4.0)  1 | 7.6(3-16.5)  6 | 17.5(8.6-31.2)  4 | **0.014** | |
| Procalcitonin (0-0.05 ng/mL)  Increased (n) | 0.03(0.02-0.05)  6 | 0.029(0.026-0.031)  1 | 0.03(0.02-0.05)  3 | 0.07(0.03-0.16)  2 | 0.207 | |
| D-dimer (0-0.55 mg/L)  Increased (n) | 0.2(0.1-0.4)  4 | 0.1 (0-0.3)  1 | 0.2(0.1-0.3)  1 | 0.6(0.4-1.2)  2 | **0.045** | |
| Erythrocyte sedimentation rate (0–20 mm/h)  Increased (n) | 22.0(10-46)  13 | 17.3(5.0-22.5)  3 | 18.0(9.3-34)  6 | 67.6(40.5-105.3)  4 | **0.025** | |
| Fibrinogen content (1.8-3.5 g/L)  Increased (n) | 2.9(2.5-3.7)  6 | 2.7(2.0-3.0)  0 | 2.8(2.5-3.5)  3 | 4.9(3.6-5.3)  3 | **0.012** | |
| Total protein (TP, 60-82 g/L) | 74.8(70.2-78.1) | 73.4(69.2-80.0) | 74.8(70.7-78.0) | 73.4(70.1-77.6) | 0.969 | |
| Albumin (34-48 g/L) | 43.3(40.0-47.2) | 46.5(42.0-47.4) | 43.8(40.1-47.1) | 38.1(35.4-41.7) | 0.052 | |
| Creatine kinase (40-200 U/L)  Increased (n) | 86.3 (54.8-123.1)  3 | 81.5 (40.0-104.6)  0 | 95.2 (57.8-172.5)  2 | 89.8(50.9-224.5)  1 | 0.546 | |
| Alanine aminotransferase (5-40 U/L)  Increased (n) | 19.4 (11.7-32.2)  1 | 12.0 (9.0-30.3)  0 | 24.1 (11.7-36.0)  1 | 20.3(18.5-29.3)  0 | 0.439 | |
| Aspartate transferase (8-35 U/L)  Increased (n) | 21.8 (18.2-27.3)  2 | 24.7 (15.7-28.5)  1 | 21.1 (18.4-26.0)  0 | 24.7(19.7-38.6)  1 | 0.631 | |
| Gamma-glutamyltransferase (7-32 U/L)  Increased (n) | 27.7 (16.4-44.2)  9 | 16.4 (15.0-36.8)  2 | 29.6 (18.9-42.9)  5 | 39.9(24.4-62.7)  2 | 0.148 | |
| Hydroxybutyrate dehydrogenase (72-182 U/L)  Increased (n) | 169.0(155.0-215.1)  9 | 155.0(127.0-163.5)  0 | 181.8(166.0-250.8)  6 | 211.3(165.9-257.0)  3 | **0.015** | |
| Lactic dehydrogenase (120-250 U/L)  Increased (n) | 188.7(166.4-238.3)  4 | 166.4(151.9-185.0)  0 | 205.5 (174.3-254.1)  3 | 218.3(175.0-285.7)  1 | **0.026** |  |
| ***Radiography (%)*** |  |  |  |  |  | |
| Pulmonary ground-glass opacities | 7(30.4) | 0 | 3(25.0) | 4(100.0) | **0.002** | |
| ***Incidence*** |  |  |  |  |  | |
| **Median time interval between first visit (IQR)** | 1.0(0-3.0) | 0(0-1.0) | 1.0(0-4.0) | 1.0(0.25-14.5) | 0.251 | |
| **Median time interval between diagnosis (IQR)** | 2.0(1-3.0) | 0(0-1.0) | 2.0(2.0-3.0) | 1.5(0.25-7.25) | **0.007** | |
| **Become SARS-CoV-2 PCR-negative, days (IQR)** | 12.0(10.0-16.0) | 12.0(10.0-15.0) | 12.5(11.0-15.8) | 18.0(7.5-26.3) | 0.604 | |
| **Outcome (%)**  Transferred to the intensive care unit  Relapse | 2(8.7)  6(26.1) | 0  2(28.6) | 0  4(33.3) | 2(50.0)  0 | **0.006**  0.415 | |

**Table S2. Demographic** and clinical features of inpatients with influenza stratified by different viruses

| **Characteristics** | **Total (n=74)** | **Influenza A (n=59)** | **Influenza B (n=15)** | ***P* Value** |
| --- | --- | --- | --- | --- |
| **Sex (%)**  Female  Male | 37(50.0)  37(50.0) | 31(52.5)  28(47.5) | 6(40.0)  9(60.0) | 0.564 |
| **Age, years (IQR)**  **Age, groups (%)**  0-14 years  15-49 years  50-64 years  ≥65 years | 45.5(28.8-58.0)  4(5.4)  37(50.0)  25(33.8)  8(10.8) | 46.0(29.0-58.0)  3(5.1)  30(50.8)  19(32.2)  7(11.9) | 34.0(21.0-57.0)  1(6.7)  7(46.7)  6(40.0)  1(6.7) | 0.435  0.894 |
| **Underlying disease (%)**  None  1 type  2 types  3 types | 35(47.3)  19(25.7)  15(20.3)  5(6.8) | 27(45.8)  15(25.4)  13(22.0)  4(6.8) | 8(53.3)  4(26.7)  2(13.3)  1(6.7) | 0.897 |
| ***Symptoms (%)*** |  |  |  |  |
| Fever | 65(87.8) | 53(89.8) | 12(80.0) | 0.375 |
| Cough | 73(98.6) | 59(100) | 14(93.3) | 0.203 |
| Sputum production | 64(86.5) | 51(86.4) | 13(86.7) | 0.982 |
| Weakness | 8(10.8) | 5(8.5) | 3(20.0) | 0.347 |
| Shortness of breath | 28(37.8) | 22(37.3) | 6(40.0) | 0.847 |
| Sore muscles | 4(5.4) | 1(1.7) | 3(20.0) | **0.025** |
| Sore throat | 12(16.2) | 10(16.9) | 2(13.3) | 0.734 |
| Headache | 15(20.3) | 10(16.9) | 5(33.3) | 0.169 |
| Chest pain | 5(6.8) | 5(8.5) | 0 | 0.576 |
| Chills | 25(33.8) | 19(32.2) | 6(40.0) | 0.559 |
| Nasal obstruction | 8(10.8) | 6(10.2) | 2(13.3) | 0.660 |
| Runny nose | 6(8.1) | 6(10.2) | 0 | 0.337 |
| Nausea | 7(9.5) | 4(6.8) | 3(20.0) | 0.143 |
| Vomiting | 7(9.5) | 5(8.5) | 2(13.3) | 0.624 |
| Diarrhea | 5(6.8) | 4(6.8) | 1(6.7) | 0.988 |
| ***Body signs (at admission, IQR)*** |  |  |  |  |
| Body temperature (>37℃) | 24(32.4) | 20(33.9) | 4(26.7) | 0.761 |
| Pulse rate (>100 per minute) | 21(28.4) | 19(32.2) | 2(13.3) | 0.206 |
| Respiratory rate (>20 per minute) | 20(27.0) | 17(28.8) | 3(20.0) | 0.746 |
| Blood pressure (SBP>140mmHg or DBP>90mmHg) | 18(24.3) | 16(27.1) | 2(13.3) | 0.333 |
| Duration of fever after admission, days (IQR) | 1.0(0-2.3) | 1.0(0-2.0) | 1.0(0-3.0) | 0.956 |
| ***Complications (%)*** |  |  |  |  |
| **Infection with other respiratory pathogens**  *Mycoplasma pneumoniae*  *Legionella pneumophila*  *Rickettsia* | 3(4.1)  1(1.4)  2(2.7) | 1(1.7)  1(1.7)  1(1.7) | 2(13.3)  0  1(6.7) | 0.136 |
| **Bronchitis** | 5(6.8) | 4(6.8) | 1(6.7) | 0.988 |
| **Abortion** | 4(5.4) | 4(6.8) | 0 | 0.576 |
| **Hepatic or renal insufficiency** | 13(17.6) | 9(15.3) | 4(26.7) | 0.446 |
| ***Laboratory tests (reference values, IQR)*** |  |  |  |  |
| White blood cells (4-10 × 10⁹ cells per L)  Increased (n) | 7.7(4.8-9.8)  18 | 7.2(4.6-9.7)  14 | 8.8(6.9-10.0)  4 | 0.104 |
| Neutrophile granulocyte (1.8-6.3 × 10⁹ cells per L)  Increased (n) | 4.1(2.8-7.0)  25 | 4.1(2.8-7.0)  20 | 4.9(3.1-7.1)  5 | 0.510 |
| Neutrophilic granulocyte percentage (40-75%)  Increased (n) | 70.6(53.4-81.2)  25 | 72.0(59.2-81.4)  21 | 54.9(43.4-78.0)  4 | 0.146 |
| Lymphocytes (1·1-3·2 × 10⁹ cells per L)  Decreased (n) | 1.3(0.9-1.8)  30 | 1.1(0.9-1.6)  26 | 2.2(0.9-3.6)  4 | **0.030** |
| Lymphocyte percentage (20-50%)  Decreased (n) | 19.4(12.3-35.0)  39 | 18.6(12.0-30.9)  35 | 24.9(12.5-43.2)  4 | 0.146 |
| Red blood cells (3.5-5 × 10^12^ cells/L)  Decreased (n) | 3.8(3.5-4.8)  20 | 3.8(3.4-4.7)  17 | 4.3(3.6-5.1)  3 | 0.259 |
| Hemoglobin (110-150g/L)  Decreased (n) | 106.6(82.7-128.4)  40 | 106.2(81.5-128.3)  34 | 117.0(92.0-130.0)  6 | 0.300 |
| Hematocrit (Hct, 35-45%)  Decreased (n) | 33.1(27.4-39.6)  45 | 32.3(26.5-39.6)  37 | 34.1(31.1-39.9)  8 | 0.353 |
| Platelet count (PLT, 100-300 × 10⁹ cells per L)  Increased (n) | 227.5(178.0-279.2)  11 | 227.5(178.0-278.7)  7 | 228.0(177.0-310.0)  4 | 0.652 |
| C-reactive protein (0-6 mg/L)  Increased (n) | 25.6(13.8-66.8)  64 | 25.0(15.8-85.4)  52 | 26.1 (11.8-54.1)  12 | 0.717 |
| Procalcitonin (0-0.05 ng/mL)  Increased (n) | 0.21(0.04-0.54)  54 | 0.23(0.04-0.57)  44 | 0.10(0.03-0.40)  10 | 0.261 |
| D-dimer (0-0.55 mg/L)  Increased (n) | 0.65(0.42-2.2)  45 | 0.61(0.42-2.2)  35 | 0.70(0.40-2.20)  10 | 0.609 |
| Erythrocyte sedimentation rate (0–20 mm/h)  Increased (n) | 36.5(21.8-78.5)  57 | 36.5(22.0-86.0)  47 | 42.0 (20.0-76.0)  10 | 0.888 |
| Fibrinogen content (1.8-3.5 g/L)  Increased (n) | 3.7 (3.1-4.8)  38 | 3.5(2.8-4.8)  28 | 4.0(3.2-7.0)  10 | 0.170 |
| Total protein (TP, 60-82 g/L)  Decreased (n) | 65.6(59.1-71.2)  20 | 64.7(58.2-68.6)  20 | 70.4(65.4-74.0)  0 | **0.015** |
| Albumin (34-48 g/L)  Decreased (n) | 32.6(30.3-38.0)  42 | 31.9(30.3-37.5)  35 | 35.2(31.6-39.2)  7 | 0.179 |
| Alanine aminotransferase (5-40 U/L)  Increased (n) | 19.4(12.4-32.4)  10 | 17.0(12.1-31.0)  4 | 24.0 (17.8-55.0)  6 | **0.045** |
| Aspartate transferase (8-35 U/L)  Increased (n) | 29.0(24.5-43.5)  29 | 29.0(25.0-42.0)  23 | 31.0 (18.0-53.0)  6 | 0.798 |
| Gamma-glutamyltransferase (7-32 U/L)  Increased (n) | 29.0(18.0-63.5)  32 | 27.0(16.0-40.3)  24 | 61.0 (22.2-108.0)  8 | 0.058 |
| Hydroxybutyrate dehydrogenase (72-182 U/L)  Increased (n) | 242.5(190.8-292.5)  57 | 230.0(182.0-271.0)  43 | 315.0(283.0-331.0)  14 | **0.000** |
| Lactic dehydrogenase (120-250 U/L)  Increased (n) | 289.0(246.0-351.8)  51 | 283.0(244.0-312.0)  37 | 358.0(302.0-387.0)  14 | **0.001** |
| ***Radiography (%)*** |  |  |  |  |
| Pulmonary consolidation and pleural effusion | 22(29.7) | 18(30.5) | 4(26.7) | 0.771 |
| ***Incidence*** |  |  |  |  |
| **Median time interval between first visit (IQR)** | 1.0(0-3.0) | 1.0(0-3.0) | 2.0(1.0-5.0) | 0.142 |
| **Median time interval between diagnosis (IQR)** | 3.0(1.0-5.0) | 3.0(1.0-5.0) | 4.0(3.0-15.0) | **0.042** |
| **Outcome (%)**  Transferred to the intensive care unit  Death | 8(10.8)  1(1.4) | 8(13.6)  0 | 0  1(6.7) | 0.195  0.203 |

**Table S3. Laboratory examinations and imaging characteristics at admission in patients with COVID-19 or influenza-A**

| **Characteristics** | **Total (n=82)** | **COVID-19 (n=23)** | **Influenza A (n=59)** | ***P* Value** |
| --- | --- | --- | --- | --- |
| ***Blood routine test (IQR)*** |  |  |  |  |
| White blood cells (×10⁹/L) | 6.7(5.2-8.6) | 6.6(5.6-7.9) | 7.2(4.6-9.7) | 0.639 |
| Neutrophile granulocyte (×10⁹/L) | 4.0(2.8-6.5) | 3.7(3.0-5.0) | 4.1(2.8-7.0) | 0.254 |
| Neutrophilic granulocyte (%) | 67.0(54.6-79.6) | 56.8(49.2-64.5) | 72.0(59.2-81.4) | **0.000** |
| Lymphocytes (×10⁹/L) | 1.4(0.9-1.9) | 1.9(1.6-2.6) | 1.1(0.9-1.6) | **0.000** |
| Lymphocyte (%) | 22.5(13.1-36.0) | 33.1(25.9-40.7) | 18.6(12.0-30.9) | **0.000** |
| Red blood cells (×10^12^/L) | 4.3(3.6-4.8) | 4.7(4.4-5.1) | 3.8(3.4-4.7) | **0.001** |
| Hemoglobin (g/L) | 114.0(90.3-131.4) | 129.3(115.8-139.1) | 106.2(81.5-128.3) | **0.001** |
| Hematocrit (%) | 34.8(28.2-42.3) | 41.2(37.2-44.6) | 32.3(26.5-39.6) | **0.000** |
| Platelet (10⁹/L) | 227.8(178.0-278.7) | 245.2(176.6-280.0) | 227.5(178.0-278.7) | 0.496 |
| ***Coagulation function (IQR)*** |  |  |  |  |
| D-dimer (mg/L) | 0.55(0.23-1.43) | 0.22(0.10-0.43) | 0.61(0.42-2.17) | **0.000** |
| Fibrinogen content (g/L) | 3.4(2.5-4.7) | 2.9(2.5-3.7) | 3.5(2.8-4.8) | **0.035** |
| Erythrocyte sedimentation rate (mm/h) | 35.0(19.5-52.0) | 22.0(10.0-46.0) | 36.5(22.0-86.0) | **0.015** |
| ***Biochemical test (IQR)*** |  |  |  |  |
| Total protein (g/L) | 68.1(60.2-73.8) | 74.8(70.2-78.1) | 64.7(58.2-68.6) | **0.000** |
| Albumin (g/L) | 35.0(30.4-41.4) | 43.3(40.0-47.2) | 31.9(30.3-37.5) | **0.000** |
| Alanine aminotransferase (U/L) | 18.7(11.9-31.7) | 19.4(11.7-32.2) | 17.0(12.1-31.0) | 0.776 |
| Aspartate transferase (U/L) | 28.0(22.4-38.0) | 21.8(18.2-27.3) | 29.0(25.0-42.0) | **0.000** |
| Gamma-glutamyltransferase (U/L) | 27.3(16.2-40.3) | 27.7(16.4-44.2) | 27.0(16.0-40.3) | 0.955 |
| Hydroxybutyrate dehydrogenase (U/L) | 200.0(172.3-255.5) | 169.0(155.0-215.1) | 230.0(182.0-271.0) | **0.002** |
| Lactic dehydrogenase (U/L) | 252.0(213.7-302.8) | 188.7(166.4-238.3) | 283.0(244.0-312.0) | **0.000** |
| ***Infection and immunity (IQR)*** |  |  |  |  |
| C-reactive protein (mg/L) | 18.6(7.0-57.2) | 4.8(2.2-14.4) | 25.0(15.8-85.4) | **0.000** |
| Procalcitonin (ng/mL) | 0.08(0.03-0.44) | 0.03 (0.02-0.05) | 0.23(0.04-0.57) | **0.000** |

**Table S4. Laboratory examinations and imaging characteristics at admission in patients with COVID-19 or influenza-B**

| **Characteristics** | **Total (n=38)** | **COVID-19 (n=23)** | **Influenza B (n=15)** | ***P* value** |
| --- | --- | --- | --- | --- |
| ***Blood routine test (IQR)*** |  |  |  |  |
| White blood cells (×10⁹/L) | 6.9(5.7-8.8) | 6.6(5.6-7.9) | 8.8(6.9-10.0) | **0.010** |
| Neutrophile granulocyte (×10⁹/L) | 3.9(3.0-5.3) | 3.7(3.0-5.0) | 4.9(3.1-7.1) | 0.162 |
| Neutrophilic granulocyte (%) | 56.5(48.1-67.6) | 56.8(49.2-64.5) | 54.9(43.4-78.0) | 0.658 |
| Lymphocytes (×10⁹/L) | 1.9(1.4-2.9) | 1.9(1.6-2.6) | 2.2(0.9-3.6) | 1.000 |
| Lymphocyte (%) | 32.7(23.5-41.1) | 33.1(25.9-40.7) | 24.9(12.5-43.2) | 0.260 |
| Red blood cells (×10^12^/L) | 4.5(4.0-5.1) | 4.7(4.4-5.1) | 4.3(3.6-5.1) | 0.202 |
| Hemoglobin (g/L) | 123.8(108.8-138.8) | 129.3(115.8-139.1) | 117.0(92.0-130.0) | 0.114 |
| Hematocrit (%) | 39.2(33.9-43.3) | 41.2(37.2-44.6) | 34.1(31.1-39.9) | **0.002** |
| Platelet (10⁹/L) | 241.6(176.9-285.1) | 245.2(176.6-280.0) | 228.0(177.0-310.0) | 0.930 |
| ***Coagulation function (IQR)*** |  |  |  |  |
| D-dimer (mg/L) | 0.39(0.16-1.34) | 0.22(0.10-0.43) | 0.74(0.42-2.20) | **0.000** |
| Fibrinogen content (g/L) | 3.2(2.7-4.1) | 2.9(2.5-3.7) | 4.0(3.2-7.0) | 0.068 |
| Erythrocyte sedimentation rate (mm/h) | 23.5(13.8-51.8) | 22.0(10.0-46.0) | 42.0(20.0-76.0) | **0.001** |
| ***Biochemical test (IQR)*** |  |  |  |  |
| Total protein (g/L) | 72.7(68.7-77.8) | 74.8(70.2-78.1) | 70.4(65.4-74.0) | **0.017** |
| Albumin (g/L) | 40.3(35.1-45.2) | 43.3(40.0-47.2) | 35.2(31.6-39.2) | **0.000** |
| Alanine aminotransferase (U/L) | 20.3(12.4-37.1) | 19.4(11.7-32.2) | 24.0(17.8-55.0) | 0.137 |
| Aspartate transferase (U/L) | 24.5(18.1-32.2) | 21.8(18.2-27.3) | 31.0(18.0-53.0) | **0.048** |
| Gamma-glutamyltransferase (U/L) | 30.1(18.3-59.8) | 27.7(16.4-44.2) | 61.0(22.2-108.0) | **0.030** |
| Hydroxybutyrate dehydrogenase (U/L) | 230.3(163.1-302.5) | 169.0(155.0-215.1) | 315.0(283.0-331.0) | **0.000** |
| Lactic dehydrogenase (U/L) | 241.6(182.0-341.5) | 188.7(166.4-238.3) | 358.0(302.0-387.0) | **0.000** |
| ***Infection and immunity (IQR)*** |  |  |  |  |
| C-reactive protein (mg/L) | 11.1(2.8-24.9) | 4.8(2.2-14.4) | 26.1(11.8-54.1) | **0.013** |
| Procalcitonin (ng/mL) | 0.04(0.03-0.17) | 0.03 (0.02-0.05) | 0.11(0.03-0.36) | **0.024** |

**Table S5. Subgroups analysis between the patients with three types of COVID-19 and influenza**

| **Characteristics** | **Influenza (n=74)** | **Mild (n=7)** | ***P* Value** | **Moderate (n=12)** | ***P* Value** | **Severe (n=4)** | ***P* Value** |
| --- | --- | --- | --- | --- | --- | --- | --- |
| **Age (years, IQR)** | 45.5(28.8-58.0) | 24.0(10.0-37.0) | **0.041** | 36.0(26.0-51.0) | 0.458 | 44.0(25.3-63.5) | 0.847 |
| **Age (grous, %)**  0-14 years  15-49 years  50-64 years  ≥65 years | 4(5.4)  37(50.0)  25(33.8)  8(10.8) | 2(28.6)  4(57.1)  1(14.3)  0 | 0.102 | 0  8(66.7)  3(25.0)  1(8.3) | 0.682 | 0  2(50.0)  2(50.0)  0 | 0.817 |
| **Male (%)** | 37(50.0) | 5(71.4) | 0.278 | 6(50.0) | 1.000 | 2(50.0) | 1.000 |
| **Underlying disease (%)**  None  1 kind  2 kinds | 35(47.3)  19(25.7)  15(20.3) | 7(100.0)  0  0 | 0.068 | 9(75.0)  1(8.3)  1(8.3) | 0.294 | 3(75.0)  0  0 | 0.253 |
| 3 kinds | 5(6.8) | 0 |  | 1(8.3) |  | 1(25.0) |  |
| Fever  Cough  Sputum production | 65(87.8)  73(98.6)  64(86.5) | 3(42.9)  1(14.3)  1(14.3) | **0.002**  **0.000**  **0.000** | 11(91.7)  6(50.0)  4(33.3) | 0.701  **0.000**  **0.000** | 4(100.0)  4(100.0)  4(100.0) | 0.458  0.815  0.431 |
| Neutrophilic granulocyte percentage (40-75%) Lymphocytes (1.1-3.2 × 10⁹ cells per L)  Lymphocyte percentage (20-50%)  Red blood cells (3.5-5 × 10^12^ cells per L)  Hemoglobin (110-150g/L)  Hematocrit (Hct, 35-45%)  C-reactive protein (0-6 mg/L)  Procalcitonin (0-0.05 ng/mL)  D-dimer (0-0.55 mg/L) | 0.21(0.04-0.54)  0.65(0.42-2.2)  36.5(21.8-78.5)  3.7 (3.1-4.8)  65.6(59.1-71.2)  32.6(30.3-38.0)  29.0(24.5-43.5)  242.5(190.8-292.5)  289.0(246.0-351.8) | 0.029(0.026-0.031)  0.1 (0-0.3)  17.3(5.0-22.5)  2.7(2.0-3.0)  73.4(69.2-80.0)  46.5(42.0-47.4)  24.7 (15.7-28.5)  155.0(127.0-163.5)  166.4(151.9-185.0) | **0.003**  **0.002**  **0.009**  **0.004**  **0.005**  **0.000**  **0.045**  **0.000**  **0.000** | 0.03(0.02-0.05)  0.2(0.1-0.3)  18.0(9.3-34)  2.8(2.5-3.5)  74.8(70.7-78.0)  43.8(40.1-47.1)  21.1 (18.4-26.0)  181.8(166.0-250.8)  205.5 (174.3-254.1) | **0.003**  **0.000**  **0.011**  **0.016**  **0.000**  **0.000**  **0.002**  **0.025**  **0.000** | 0.07(0.03-0.16)  0.6(0.4-1.2)  67.6(40.5-105.3)  4.9(3.6-5.3)  73.4(70.1-77.6)  38.1(35.4-41.7)  24.7(19.7-38.6)  211.3(165.9-257.0)  218.3(175.0-285.7) | 0.204  0.571  0.157  0.257  **0.025**  0.073  0.287  0.415  **0.041** |
| Erythrocyte sedimentation rate (0–20 mm/h)  Fibrinogen content (1.8-3.5 g/L)  Total protein (TP, 60-82 g/L)  Albumin (34-48 g/L)  Aspartate transferase (8-35 U/L)  Hydroxybutyrate dehydrogenase (72-182 U/L)  Lactic dehydrogenase (120-250 U/L) | 70.6(53.4-81.2)  1.3(0.9-1.8)  19.4(12.3-35.0)  3.8(3.5-4.8)  106.6(82.7-128.4)  33.1(27.4-39.6)  25.6(13.8-66.8) | 54.7(31.9-56.3)  2.3(1.9-3.4)  36.8(32.6-53.5)  4.8(4.3-5.2)  123.7(115.8-141.9)  39.8(37.2-44.6)  2.2(1.7-4.0) | **0.012**  **0.003**  **0.008**  0.059  0.050  **0.013**  **0.000** | 56.8(50.0-62.0)  2.0(1.5-2.5)  33.8(27.1-39.5)  4.6(4.4-5.0)  137.8(122.2-139.2)  43.6(39.6-44.6)  7.6(3-16.5) | **0.010**  **0.007**  **0.005**  **0.012**  **0.005**  **0.000**  **0.000** | 71.1(60.7-72.7)  1.4(1.0-2.2)  19.2(17.8-30.3)  4.4(3.8-5.2)  115.9(102.0-126.0)  36.2(32.9-40.1)  17.5(8.6-31.2) | 0.964  0.650  0.786  0.319  0.634  0.308  0.365 |
|  |  |  |  |  |  |  |  |
|  |  |  |  |  |  |  |  |
|  |  |  |  |  |  |  |  |
|  |  |  |  |  |  |  |  |
|  |  |  |  |  |  |  |  |
